# Serum IGFBP7 levels associate with insulin resistance and the risk of metabolic syndrome in a Chinese population

**DOI:** 10.1038/srep10227

**Published:** 2015-05-18

**Authors:** Yi Liu, Minliang Wu, Jie Ling, Libing Cai, Dandan Zhang, Harvest F Gu, Hao Wang, Yimin Zhu, Maode Lai

**Affiliations:** 1 Department of Epidemiology & Biostatistics, Zhejiang University School of Public Health, Hangzhou 310058, China; 2 Department of Laboratory, the second Affiliated Hospital of Zhejiang University, School of Medicine, Hangzhou 310003, China; 3 Third Hospital of Xiaoshan, Hangzhou 311251, China; 4 Department of Pathology, Zhejiang University School of Medicine, Hangzhou 310058, China; 5 Rolf Luft Research Center for Diabetes and Endocrinology, Department of Molecular Medicine and Surgery, Karolinska Institutet, Karolinska University Hospital, Stockholm SE-171 76, Sweden

## Abstract

Metabolic syndrome (MetS), one of the major public health concerns, is regarded as the “common soil” of incidence of common chronic diseases and may increase the risk of type 2 diabetes. The predominant underlying mechanism of MetS is insulin resistance (IR). Additionally, previous studies have indicated that IGFBP7 has high affinity of binding with insulin and might induce IR. The objective of this study was to firstly evaluate the associations of serum IGFBP7 levels with IR and MetS with a relatively large sample and population based design. In a population based MetS case-control study, HOMA-IR was used to evaluate the insulin sensitivity and serum IGFBP7 levels were determined with chemiluminescence–linked immunoassay. As a result, the subjects of MetS and IR had higher serum levels of IGFBP7 than control healthy subjects. High serum IGFBP7 levels increased the risk of MetS and IR. Serum IGFBP7 levels were also found to be significantly correlated with metabolic-associated parameters of Waist-to-hip ratio (WHR), HDL and LDL. These findings suggest that serum IGFBP7 levels are associated with IR and MetS, providing new insight into the mechanism of IR and Mets. IGFBP7 may be a potential interventional target for IR and Mets.

Metabolic syndrome (MetS), which characterized as obesity, hyperglycemia, hypertension and dyslipidemia^1^,^2^,^3^, is regarded as the “common soil” of incidence of common chronic diseases and may increase the risk of type 2 diabetes, cardiovascular disease, stroke and some cancers such as colorectal, breast, pancreas, and kidney^4^,^5^,^6^. The prevalence of MetS is about 30% and varies with the definitions of MetS and populations^1^,^7^,^8^. In recent twenty years, the prevalence of MetS is increasing worldwide. Therefore, MetS and its associated chronic diseases have been one of the major public health concerns.

The predominant underlying mechanism of MetS is insulin resistance (IR)^9^. IR is defined as a reduced responsiveness of a target cell or a whole organism to the insulin concentration to which it is exposed^9^. Less efficiency to metabolism and utilization of glucose induce hyperglycemia. Since serum glucose positively regulates insulin secretion, hyperglycemia stimulates islet β cell to secret insulin, then leads to hyperinsulinemia and increases the serum IGFs (insulin-like growth factors)^10^. Continuous status of hyperinsulinemia and increased serum IGFs initiate abnormal response in cell growth, development, differentiation and metabolism^11^,^12^,^13^. Previous studies had showed high serum IGF levels were associated with MetS, type 2 diabetes and common cancers^11^,^12^,^13^,^14^. Our findings had indicated that diabetes increased the risks of common cancers including colon and rectum, lung, pancreas, liver kidney and female breast^15^,^16^.

Insulin-like growth factor binding proteins (IGFBPs) are a family of homogenous proteins and regulate the IGFs signaling pathway by binding with insulin and IGFs^17^,^18^. IGFBP 1-6 have higher ability of binding affinity to IGFs and less to insulin and play important roles on regulating cell growth via the IGFs signaling pathway^19^. However, different from the other six IGFBPs, the seventh of IGFBPs, IGFBP7, also known as IGFBP- rP1, MAC25, PSF, TAF, FSTL2 or PGI2-stimulating factor, has weak binding affinity to IGFs and relatively high affinity to insulin^20^,^21^. IGFBP7 is a 30-kDa modular secreted protein with a N-terminal domain, including 11 cysteines, a heparin binding site, a Kazal-type trypsin inhibitor domain and a C-terminal Ig-like type C repeat^19^,^20^,^22^, involved in multiple pathways mainly in an IGF- independent manner. Previous studies indicated that IGFBP7 expression was associated with tumor development and functions as tumor suppressor gene in cancers such as colon and rectum, breast, thyroid through the regulation of cell proliferation, cell adhesion, apoptosis, cellular senescence and angiogenesis^23^,^24^,^25^,^26^. IGFBP7 is also involved in TGFβ signal pathway. However, in glioma, IGFBP7 may play as an oncogenic role^27^. These studies suggested that IGFBP7 may play different roles in different cancers.

Due to the high affinity to insulin, IGFBP7 may interfere with biological response of insulin, subsequently induces IR and involves in the development of diabetes and cardiovascular diseases. Lopez-Bermejo *et al.* found increased serum IGFBP7 levels to be associated with IR^21^. Kutsukake *et al.* observed hemodialysis patients with type 2 diabetes had higher serum IGFBP7 levels than the hemodialysis patients without type 2 diabetes^28^. Recently, Gu, *et al.* found newly diagnosed type 2 diabetes had higher levels of IGFBP7 DNA methylation and low IGFBP7 may be associated with IR in type 2 diabetes^29^. The findings from these studies indicated serum IGFBP7 might be associated with IR and diabetes.

However, the sample sizes in those studies were relatively small and subjects restricted on diabetes patients. No previous study was found on the association of IGFBP7 with prediabetes such as MetS. So far, no convincing evidence was found. In our study, we had determined serum IGFBP7 levels of 1042 MetS patients and 1583 healthy control subjects from a cross-sectional survey on MetS. The objective of this study was to evaluate the associations of serum IGFBP7 with MetS and IR.

## Methods

### Subjects

A total of 2625 subjects including 1042 MetS patients and 1583 healthy control subjects of Chinese origin were recruited from XiaoShan cross-sectional survey on MetS in Hangzhou, Zhejiang Province, China in 2010. This community-based cluster sampling survey consisted of a questionnaire based epidemiological interview, a health examination and laboratory measurements. All the subjects of this investigation were Han's Chinese and aged 21 to 75 years old. The case subjects in this study were MetS patients who were diagnosed according to the criteria of Chinese Diabetes Society (CDS). The MetS criteria of CDS includes: when a subject met three or more of the following four components^17^: 1) obesity: Body Mass Index (BMI) ≥ 25 kg/m^2^; 2) fasting blood glucose (FG) ≥ 6.1 mmol/L or receiving antidiabetic medication; 3) fasting total triglyceride (TG) ≥ 1.7 mmol/L or males’ high density lipoprotein cholesterol (HDL) < 0.9 mmol/L or females’ HDL < 1.0 mmol/L; and 4) blood pressure (BP) ≥ 140/90 mmHg or receiving antihypertensive medication. Healthy control subjects were unrelated individual residents and selected from the subjects with no history of obesity, hyperglycemia, dyslipidaemia, hypertension or diabetes mellitus. Participants who had cancer, chronic diseases of the heart, liver, lung or kidney, and other endocrine diseases were excluded. All participants were given and signed the written informed consent form.

### Anthropometric measurements

Anthropometric indices, including weight, height, waist circumference (WC), hip circumference (HC) and BP were measured by well-trained investigators, following standard protocols. Height and weight were measured with the participants wearing light clothing and without shoes. WC was measured at the midpoint between the iliac crest and lowest rib. HC was measured at the widest part of the gluteal region with two feet together. BP was measured in a sitting position with a mercury sphygmomanometer after 15 minutes of rest. Systolic blood pressure (SBP) and diastolic blood pressure (DBP) were reported as the average of three repeat measurements with 30-second rest intervals between measurements. BMI was calculated as the individual's body weight in kilograms divided by the square of his or her height in meters. Waist to height ratio (WHtR) was calculated as WC in centimeters divided by height in centimeters. Waist-to-hip ratio (WHR) was calculated as HC in centimeters divided by height in centimeters.

### Biochemical measurements

After a 12-hour overnight fast, blood samples were drawn to determine the serum levels of total cholesterol (TC), TG, low density lipoprotein cholesterol (LDL), HDL, insulin and FG with a biochemical auto-analyzer (Hitachi 7060, Tokyo, Japan).

Serum IGFBP7 levels were measured by chemiluminescence–linked immunoassay, which incorporated a polyclonal and a monoclonal anti-IGFBP7 antibody (R&D Systems Inc., Abingdon, UK). The decisive coefficient (R^2^) of the standard curve was 0.9986. The average recovery rate was approximately 106.5%. The sensitivity of the assay was 0.32 ng/L.

### Statistical analysis

Quantitative data were expressed as means ± standard deviations (SD) for normal distributions, as medians (inter-quartile range) for non- normally distributed variables. Non-normally distributed variables were square-root transformed before analysis. Qualitative data were presented frequencies. Homeostasis model of assessment was used to assess insulin resistance index (HOMA-IR) and calculated as: fasting serum glucose (mmol/L) × fasting serum insulin (μU/ml)/22.5. Subjects were categorized into IR and insulin sensitive (IS) with the value of the 75^th^ percentage of HOMA-IR index in control subjects as the cut off value.

The t-test was used to compare the mean differences between two groups for normally distributed variables and the Mann-Whitney U test for non-normally distributed variables. The Chi-square test was used to compare the frequencies of differences for categorical variables. Odds ratio (OR) and 95% confident interval (95% CI) was calculated with logistic model adjusted by potential confounding factors such as age, gender and IR . Correlations between continuous variables were assessed by the spearman partial correlation coefficient (r_s_).

All statistical analyses were performed using SPSS for Windows, version 20.0 (SPSS Inc., Chicago, IL, USA). A *P*-value of <0.05 was considered to be statistically significant. The study protocol was approved by the Institutional Review Board of School of Public Health, Zhejiang University and methods were carried out in accordance with the approved guidelines.

## Results

The basic characteristics of the study population are shown in Table 1. The age (mean ± standard deviation) was 58.1 ± 9.3 years in case subjects and 52.5 ± 10.8 years in healthy controls (*P* < 0.001). 50.10% of subjects were males in MetS subjects and 47.80% in controls (*P* = 0.231). Therefore, the following analyses were carried out with adjustment of age and sex. MetS subjects had higher levels of BMI, WC, WHtR, HC, WHR, SBP, DBP, TC, TG, LDL, FG and fasting insulin than controls (all *P* < 0.001), and lower level of HDL (*P* < 0.001).

The distributions of serum IGFBP7 levels of the subjects by MetS and IR status are presented in Table 2. The median of serum IGFBP7 level in MetS patients was 45.80 ng/ml, which was significantly higher than that in healthy controls (35.80 ng/ml) (*P* < 0.001). Serum IGFBP7 levels were categorized into two groups (high and low groups) with the value of the 50^th^ percentage in control subjects (35.80 ng/ml) as the cut off value. High serum IGFBP7 levels (≥35.80 ng/ml) were associated with a significantly increased risk of MetS with the adjusted OR of 2.758 (95% CI = 2.308 – 3.297) (Table 3). After additional adjustment with BMI, association of IGFBP7 with MetS remained significant with the OR of 3.993 (3.037, 5.250).

IR status was evaluated with HOMA-IR index and categorized into IR and IS with the value of the 75^th^ percentage of HOMA-IR index in control subjects (0.98) as the cut off value. The median of serum IGFBP7 levels in IR subjects was 40.45 ng/ml, which was significantly higher than that in control subjects (38.25 ng/ml) (*P* < 0.001) (Table 2). High serum IGFBP7 levels (≥35.80 ng/ml) were associated with a significantly increased risk of IR (OR = 1.240, 95% CI = 1.055 − 1.458) (Table 3). However, the significance with IR was not found after additional adjustment with BMI.

In order to analyze the relationship among these three things (IGBP7, MetS case/ control status, and IR), we calculated the association of IGFBP7 with MetS modified by HOMA-IR, age, sex, the significance between IGFBP7 and MetS still remained with the OR of 3.497 (95%CI: 2.796-4.375).

Table 4 shows the correlations of serum IGFBP7 levels and metabolic related components. In healthy control subjects, after adjusted by sex and age, serum IGFBP7 levels were found to be negatively correlated with WC (r_s_ = −0.138, *P* < 0.001), WHtR (r_s_ = −0.151, *P* < 0.001), WHR (r_s_  = −0.137, *P* < 0.001), SBP (r_s_ = −0.086, *P* = 0.001), DBP (r_s_ = −0.071, *P* = 0.005), HDL (r_s_ = −0.121, *P* < 0.001), and positively with LDL (r_s_ =0.164, *P* < 0.001). After additional adjustment with HOMA-IR, serum IGFBP7 levels still negatively correlated with WC (r_s_ = −0.103, *P* < 0.001), WHtR (r_s_ = −0.117, *P* < 0.001), WHR (r_s_ = −0.109, *P* < 0.001), SBP (r_s_ = −0.060, *P* = 0.017), DBP (r_s_ = −0.051, *P* = 0.043), HDL (r_s_ = −0.114, *P* < 0.001), and positively with LDL (r_s_ = 0.157, *P* < 0.001). In MetS subjects the negative correlations were observed between serum IGFBP7 level with WHR (r_s_ = −0.080, *P* = 0.012) and HDL (r_s_ = −0.116, *P* < 0.001), and the positive correlation between serum IGFBP7 level with LDL (r_s_ = 0.157, *P* < 0.001) adjusted by sex and age and WHR ( r_s_ = −0.072, *P* = 0.023), HDL ( r_s_ = −0.117, *P* < 0.001) and LDL( r_s_ = 0.156, *P* < 0.001) adjusted by age, sex and HOMA-IR.

## Discussion

In present study, we determined serum IGFBP7 levels in 1042 MetS patients and 1583 healthy control subjects and found that the subjects with MetS and IR had higher levels of serum IGFBP7 than controls. Higher IGFBP7 increased the risk of MetS and IR. Serum IGFBP7 levels were also associated with some metabolic-associated parameters. The findings indicated that increased serum IGFBP7 levels might be one of the mechanisms of IR and MetS.

IGFBP7, also known as IGFBP-related protein 1, is a secreted glycoprotein and widely expressed in various human tissues^20^, including gastrointestinal tract, brain, lung and prostate. IGFBP7 has been reported to be associated with tumor development and survival although the results were not always consistent^30^,^31^,^32^. DNA methylation was regarded as the main mechanism in abnormal expression of IGFBP7^25^. In 2010, Huang *et al.*^33^ found the SNPs in the promoter-regulatory region might affect the gene expression level and modify the cancer susceptibility.

There were some previous researches intended to examine association of IGFBP7 with IR and diabetes, however the findings were not consistence. In 2004, positive correlation were found between serum IGFBP7 levels and fasting glucose^34^; In 2006, Lopez-Bermejo *et al.*^21^ firstly found that higher serum levels of circulating IGFBP7 decreased insulin sensitivity and adiponectin as well as increased C-reactive protein and soluble tumor necrosis factor receptor 2. These findings implicated that IGFBP7 might associate with IR. However, the sample in his study was relatively small (113 nondiabetic men and 43 type 2 diabetic men) and this result was not reported to be validated in the latter study. The difference might be due to selection criteria of type 2 diabetes patients and sample size. With large sample size and the samples of MetS from a cross-sectional study, we used MetS (diagnosed based on the criteria of CDS) and IR (determined as HOMA-IR), which are the prediabetes status, as the outcome of analysis. We found the subjects with MetS and IR (determined as HOMA-IR) had higher levels of serum IGFBP7. Higher serum levels of IGFBP7 increased the risks of IR and MetS.

Unlike IGFBPs 1–6, IGFBP7 has a relatively low affinity for IGF, while its affinity for insulin is 500-fold higher^19^,^20^. With insulin receptor binding assays, Yamanaka *et al.* found IGFBP7 inhibited the specific binding of ^125^I-insulin to human placental insulin receptors at 60% by 100 pmol, and 90% by 300 pmol^19^. This finding indicated IGFBP7 could compete with insulin receptors for binding of insulin and reflected higher affinity of IGFBP7 for insulin. IGFBP7 might also take effect by involving in insulin receptor autophosphorylation and IRS-1 phosphorylation^19^. From molecular structural feature perspective^18^,^19^, the insulin binding site is at the NH_2_ terminus of the IGFBP molecule, and disulfide bonds between the NH_2_ and COOH termini result in a ternary structure for the IGFBPs that confers high affinity for IGFs. IGFBPs 1-6 had the conserved NH_2_-terminal and COOH-terminal sequences and the appropriate ternary structures formed by disulfide bonds, resulting in high affinity for IGFs but low affinity for insulin. Compared with IGFBPs 1-6, IGFBP7 lacked the ternary structure, and its insulin binding site at the NH_2_ terminus was exposed, contributed to increased affinity for insulin and diminished affinity for IGFs.

All previous observations indicated that increased IGFBP7 might bind serum insulin and substantially reduced serum level of free insulin^35^, competitively inhibit the binding of insulin and IR, diminish or eliminate biological effects of insulin, and then resulting in IR. From population level, our findings supported this hypothesis.

So far, there was no report about the direct relationship between IGFBP7 and MetS. MetS is represented by a group of interrelated disorders, including central obesity, hypertension, derangement of glucose and lipid metabolism. Our results also indicated that IGFBP7 significantly associated with MetS after adjustment with age, sex (or IR). It also correlated with metabolic related components (BMI, WC, WHtR, WHR) irrespectively of adjustment of HOMA-IR. Together with these evidences, these findings indicated that IGFBP7 was an independent predictor for MetS and its effect on IR might be mediated by obesity. These findings indicated IGFBP7 might interfere the metabolism serum lipids and energy. Bonnet reported that postnatal overgrowth might be related to a dosage effect of the IGFBP7 gene^36^. Further, interactions of IGFBP7 with the IGF system possibly resulted in hyperinsulinemia^21^,^37^. More importantly, IR may lie at the heart of the MetS^38^, differentially contributing to MetS phenotype^39^.

Serum IGFBP7 was also associated with the other metabolic components. The associations of serum IGFBP7 were firstly documented with WHR, HDL and LDL both in the subjects of Mets and control and adjusted by sex, gender or additional HOMA-IR. We also found significant associations with WC, WHtR, SBP, DBP in control subjects. BMI and DBP had been found to be significant in previous study^28^,^40^. These associations have been modified potential confounders such as age, gender or additional HOMA-IR. Although the mechanisms of these associations remain uncover, these findings indicate IGFBP7 might associated the multiple biological effects and it may be an interventional target for multiple metabolic alterations. Based our previous studies, we have found several small molecular disruptors could reduce the capability of combination of IGFBP7 with insulin and then lessen IR status (unpublished data). These findings might provide a new strategy for blocking the progression of Mets and diabetes and improving metabolic condition.

After adjustment of age, sex and HOMA-IR, IGFBP7 also associated with MetS. This result indicated that IGFBP7 might be an independent predictor for MS. Together with the evidences with the associations of IGFBP7 with metabolic components, These findings indicated that IGFBP7 independently associated with MetS and its biological effects might be mediated by obesity, LDL and HDL etc.

In summary, we found that serum IGFBP7 levels are associated with IR and MetS and indicated that increased serum IGFBP7 might be one of circulating mechanism of MetS. IGFBP7 might be a new interventional target for IR and Mets.

## Additional Information

**How to cite this article**: Liu, Y. *et al*. Serum IGFBP7 levels associate with insulin resistance and the risk of metabolic syndrome in a Chinese population. *Sci. Rep.* **5**, 10227; doi: 10.1038/srep10227 (2015).

## Figures and Tables

**Table 1:** Basic characteristics of MetS and control subjects.

**Variable**	**Case (n = 1042)**	**Control (n = 1583)**	***P*****-value**
Age (year)	58.1 ± 9.3	52.5 ± 10.8	<**0.001**§
Gender (n, %)			0.231#
Male	522 (50.10%)	756 (47.80%)	
Female	519 (49.90%)	827 (52.20%)	
BMI (kg/m^2^)	27.21 ± 2.63	22.31 ± 2.50	<**0.001**§
WC (cm)	90.35 ± 7.92	78.20 ± 7.88	<**0.001**§
WHtR	0.56 ± 0.05	0.49 ± 0.05	<**0.001**§
HC (cm)	99.96 ± 5.83	92.15 ± 5.50	<**0.001**§
WHR	0.91 ± 0.06	0.85 ± 0.06	<**0.001**§
SBP (mmHg)	156.61 ± 16.52	125.64 ± 16.67	<**0.001**§
DBP (mmHg)	92.10 ± 10.08	75.71 ± 10.59	<**0.001**§
TC (mmol/L)	5.02 ± 0.92	4.39 ± 0.81	<**0.001**§
TG (mmol/L)	2.48 (1.99, 3.41)	1.23 (0.93, 1.52)	<0.001*
HDL (mmol/L)	1.38 (1.18, 1.62)	1.63 (1.39, 1.96)	<0.001*
LDL (mmol/L)	2.22 ± 0.75	1.81 ± 0.56	<**0.001**§
FG (mmol/L)	5.70 (5.04, 6.68)	4.89 (4.59, 5.24)	<0.001*
Insulin (μU/ml)	5.63 (3.98, 7.51)	3.19 (2.32, 4.39)	<0.001*
HOMA-IR index	1.46 (1.01, 2.00)	0.70 (0.50, 0.98)	<0.001*

Quantitative data were presented as mean ± standard deviation for age, BMI, WC, WHtR, HC, WHR, SBP, DBP,TC and LDL, and median (P25, P75) for TG, HDL, FG, insulin, HOMA-IR.

BMI = weight/height^2^, WHtR = WC/height, WHR = WC/HC, FG = fasting blood glucose HOMA-IR index = insulin × FG/22.5

^§^independent t-test.

^#^χ^2^ test.

^*^rank sum test.

**Table 2:** Serum IGFBP7 levels in the subjects by MetS and IR status.

**Group**	**N**	**Median**	**P**_**25**_	**P**_**75**_	***P*****-value**#
MetS					
Control	1575	35.80	28.70	46.40	<0.001
Case	1040	45.80	36.50	59.05	
					
HOMA-IR index§					
<0.98	1420	38.25	30.30	50.53	<0.001
≥0.98	1192	40.45	31.83	52.45	

^#^P values calculated with the Mann-Whitney U test.

^§^categorized by the value of 75^th^ percentage in control group.

**Table 3:**
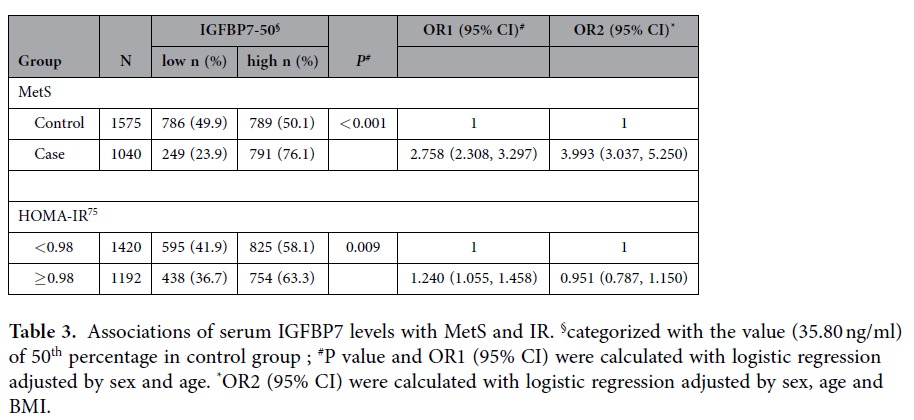
Associations of serum IGFBP7 levels with MetS and IR.

**Table 4:** Correlations between serum IGFBP7 levels and metabolic-associated parameters.

	**Control**					**Case**		
	**r**_**s**_§	**P**§	**r**_s_*	P*	**r**_**s**_§	**P**§	**r**_s_*	P*
WC (cm)	−0.138	<**0.001**	−0.103	<**0.001**	−0.056	0.078	−0.041	>0.10
WHtR	−0.151	<**0.001**	−0.117	<**0.001**	−0.074	>0.10	−0.059	0.064
HC (cm)	−0.079	**0.002**	−0.047	0.067	−0.003	>0.10	0.011	>0.10
WHR	−0.137	<**0.001**	−0.109	<**0.001**	−0.080	**0.012**	−0.072	**0.023**
BMI (kg/m2)	−0.085	**0.001**	−0.041	>0.10	−0.013	>0.10	0.005	>0.10
SBP (mmHg)	−0.086	**0.001**	−0.060	**0.017**	0.050	0.108	0.055	0.080
DBP (mmHg)	−0.071	**0.005**	−0.051	**0.043**	0.040	>0.10	0.041	>0.10
TC (mmol/L)	0.011	>0.10	0.024	>0.10	0.050	0.109	0.055	0.078
TG (mmol/L)^#^	−0.063	**0.013**	−0.032	>0.10	0.009	>0.10	0.018	>0.10
HDL (mmol/L)^#^	−0.121	<**0.001**	−0.114	<**0.001**	−0.116	<**0.001**	−0.117	<**0.001**
LDL (mmol/L)	0.164	<**0.001**	0.157	<**0.001**	0.157	<**0.001**	0.156	<**0.001**
FG(mmol/L)^#^	−0.065	**0.010**	−0.009	>0.10	−0.013	>0.10	0.017	>0.10
Insulin (μU/ml)^##^	−0.127	<**0.001**	0.009	>0.10	−0.064	**0.040**	−0.017	>0.10
HOMA−IR^#^	−0.132	<**0.001**			−0.063	**0.044**		

^§^Spearman correlation adjusted by sex and age.

^*^Spearman correlation adjusted by sex, age and HOMA-IR.
